# Measuring processes of change in behavioural interventions: Insights gained from linking mechanisms of action to associated measures

**DOI:** 10.1111/bjhp.70015

**Published:** 2025-08-18

**Authors:** Paulina M. Schenk, Susan Michie, Marie Johnston, Talea Cornelius

**Affiliations:** ^1^ Centre for Behaviour Change University College London London UK; ^2^ Aberdeen Health Psychology Group University of Aberdeen Aberdeen UK; ^3^ Center for Behavioral Cardiovascular Health Columbia University Irving Medical Center New York New York USA

**Keywords:** behaviour, measure, measurement, mechanism, ontology

## Abstract

**Background:**

Testing mechanisms of action (MoAs) hypothesized to drive behaviour change improves intervention efficacy and allows theoretical propositions to be evaluated, enabling evidence accumulation. However, clear correspondence between behavioural MoAs and associated measures is lacking, creating challenges for intervention evaluation.

**Aims:**

To link well‐defined behavioural MoAs to multidimensional measures.

**Method:**

Two researchers independently judged whether 44 measures (comprising 131 (sub)scales) in the Science of Behavior Change (SOBC) Measures Repository are suitable for measuring 270 MoAs from the Human Behaviour‐Change Project's MoA Ontology (2022). Links were categorized as ‘confirmed’ (aligned with a prior expert opinion study linking measures to 26 MoAs), ‘removed’ or ‘new’. Judgements were compared, discussed, reconciled iteratively and jointly reviewed for consistency.

**Results:**

Six hundred and eighty‐six links between SOBC measures and MoAs were identified (397 ‘confirmed’, 289 ‘new’). Measures were found to tap into multiple MoAs, with 5.24 MoAs linked to each measure on average. These links demonstrated greater granularity than those identified in a previous expert opinion study because MoAs from the MoA Ontology were more specific than MoAs from this previous study. Commonly co‐occurring MoAs were identified (e.g., ‘self‐regulation process’ and ‘self‐regulation capability’) and MoAs potentially missing from the ontology were noted.

**Conclusion:**

The refined measure‐MoA links provide more precise guidance for researchers when designing and/or selecting measures to assess the role of MoAs in theory‐based behavioural interventions. Future research should further explore measure‐MoA links by, for example, testing the discriminant content validity of ostensibly distinct measures that tap into the same or similar MoAs.

## INTRODUCTION

Understanding the mechanisms by which behavioural interventions elicit desired change (e.g., decreased sedentary time, increased medication adherence) has the potential to increase their potency and scalability. As described by the Science of Behavior Change (SOBC) program (Sumner et al., 2018, 2019), the experimental medicine approach to behaviour change necessitates that a researcher (1) identifies a putative mechanism that underlies behaviour change, (2) measures that mechanism, (3) tests whether an intervention leads to a change in the measured mechanism and, finally, (4) tests whether change in the mechanism is associated with a change in behaviour. Although the value of this approach is widely recognized (Riddle, 2015), few randomized trials actually measure a hypothesized mechanism, and even fewer test whether mechanism change underlies behaviour change (Edmondson et al., 2018). Furthermore, there is not enough research that matches measures to their proposed *mechanisms of action* (*MoAs*) (see glossary of bold and italicized terms in Table 1), and many measurements and their subscales are complex and multi‐dimensional, meaning they do not comprehensively assess a specific MoA. Without a clear link between the measurements used and the MoAs that they tap into, there are significant challenges for behavioural scientists working to refine theories and develop robust, theory‐based interventions. To support the testing of interventions' MoAs, the SOBC has provided resources to behavioural scientists, including an online *repository* comprising putative measures of MoAs (https://measures.scienceofbehaviorchange.org/).

**TABLE 1:** Glossary of terms.

Label	Definition	References
Mechanism of action (MoA)	A process through which an intervention (e.g., a behaviour change intervention) influences an outcome behaviour	Schenk et al. (2024)
Repository	A centralized data storage in which files and resources can be kept	Gupta (2023)
Ontology	A classification framework that includes representations of entities, as well as unique labels, definitions and alphanumeric IDs for these entities and specifies the relationships between entities	Arp et al. (2015)
Entity	Anything that exists, including processes, objects and their objects. Entities are represented in ontologies, and these representations are referred to as ‘class’	Arp et al. (2015)
Class	In ontologies, classes represent types or groupings of entities in the world. The terms ‘entity’ and ‘class’ are sometimes used interchangeably when referring to representations of entities in an ontology. Ontologies are hierarchically structure by linking a class to its parent class (see definition of parent class)	Arp et al. (2015)
Parent class	A class that has a hierarchical relationship to one or more subclasses, with the subclasses having all the properties of the parent and an additional more specific property	Arp et al. (2015)
Relationship	The way in which two things (e.g., classes) are linked	Arp et al. (2015)

A complementary effort to the SOBC has been led by the Human Behaviour Change Project's researchers (HBCP; Michie et al., 2017; Michie et al., 2020; Wright et al., 2020), who focused on clearly operationalizing and organizing MoAs commonly targeted in these behavioural interventions. Clear operationalization of these MoAs is a critical component of conducting valid mechanistic hypothesis tests and a necessary precursor to implementing robust measurement (i.e., one must operationalize a construct before it is possible to validate and utilize a measure of that construct) (Derby et al., 2024). As a first step in addressing this gap, in a collaborative project between SOBC and the HBCP, we conducted an expert opinion study to identify links between self‐report measures in the online SOBC Measures Repository (Science Of Behavior Change (SOBC)) and 26 MoAs identified from behavioural theories and models (Bohlen et al., 2019; Carey et al., 2019; Connell et al., 2019; Johnston et al., 2018, 2021). This study identified 167 measure‐MoA links (Cornelius et al., 2023); however, the 26 MoAs included in this study comprised broad categories such as ‘Memory, Attention and Decision‐Making’ that did not capture more granular MoAs or less frequently investigated but potentially influential MoAs in behaviour change interventions. This limitation meant that the mapping cannot be used to precisely identify which specific MoAs each measurement taps into. For instance, the ‘Cognitive Reflection Test’ includes items related to self‐regulation and cognitive processes. To capture its cognitive aspects, the broad ‘Memory, Attention and Decision‐Making’ MoA was linked to this measurement. However, this link provides a general overview of the MoAs (e.g., cognitive process) associated with the ‘Cognitive Reflection Test’, which may limit its utility for users of the mapping.

**FIGURE 1:**
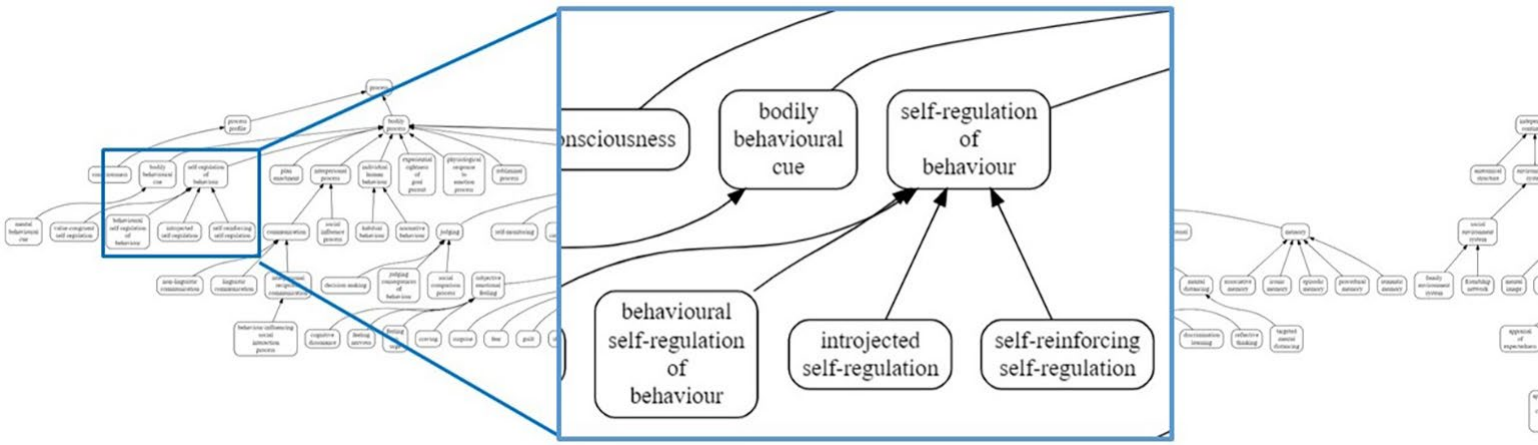
Snapshot of the Mechanism of Action (MoA) Ontology in its hierarchical format, with the arrows showing parent class–class relationships (e.g., self‐regulation of behaviour with its subclasses).

More recently, the HBCP has undertaken a mammoth project to define and organize a greatly expanded set of MoAs hypothesized to underlie behaviour change into a sub‐ontology as part of its Behaviour Change Intervention Ontology (BCIO): The MoA Ontology (Michie et al., 2017, 2020; Schenk et al., 2024). This ontology's goal is to provide a clear and extensive classification system that labels, defines and organizes MoAs in behaviour change intervention research. To capture a wide range of MoAs and reflect various perspectives on MoAs, the MoA Ontology was developed by drawing on the constructs of 83 behavioural theories, applying the ontology to code MoAs in various published intervention reports and consulting international behavioural science experts (Schenk et al., 2024). This ontology was iteratively updated, with the published version including 284 *classes* (i.e., groupings or categories) of MoAs organized onto seven hierarchical levels. An example of the structure of the ontology's hierarchy can be seen in Figure 1. The ontology has since been further updated to more precisely capture the constructs of behavioural theories, now including 621 classes to capture MoAs (release date: 18 July 2025). The most up‐to‐date version of the ontology will always be available on the Human Behaviour Change Project's GitHub repository: https://github.com/HumanBehaviourChangeProject/ontologies/tree/master/MechanismOfAction.

Ontologies are formally defined as classification systems that include classes (representations of *entities*, anything that exists in the universe, including objects, processes and attributes) with unique IDs, labels and definitions and relationships (Arp et al., 2015). These are often organized in a hierarchical format, with classes nested under a *parent class*, and each class definition should be unambiguous, clearly specifying a class's parent class and its distinguishing attributes from that parent class (Michie et al., 2019; Seppälä et al., 2017). For example, the definition of the class ‘motivation’ within the MoA Ontology first specifies that it is a ‘mental process’ (parent class) and then describes what distinguishes motivation from other subclasses belonging to the ‘mental process’ parent class. Ontologies are developed to be readable by both humans and computers, meaning they can be used to develop reasoning algorithms for applications such as data extraction and prediction about their classes and *relationships* (Hastings, 2017). Each class in an ontology has a unique alphanumeric ID (e.g., BCIO:006133), enabling them to be computer readable. They are also intended to be updated based on evidence and user feedback (He et al., 2018), so that they become increasingly useful and/or remain relevant resources for the research community over time.

The expert opinion study linking SOBC measurements to MoAs (Cornelius et al., 2023) was limited by the inclusion of only 26 MoAs, as well as the use of the term ‘related’ to determine measure–MoA links (i.e., it was not necessary that the measure actually assessed these MoAs to create a link). Furthermore, the SOBC measures were considered as a unit, with no recognition of potentially different MoAs across subscales. The present study was undertaken with the goal of improving the utility and specificity of this previous mapping work. Using the 167 measure–MoA links as a starting point (Cornelius et al., 2023), we aimed to confirm, remove and create new links between the measure subscales, with a focus on whether items in these scales actually intended to assess different classes of MoAs in the expanded MoA Ontology. We anticipated that the results of this study would provide valuable information regarding the content of these measures for assessing different MoAs, as well as data regarding potential overlap in assessment tools between definitionally non‐overlapping classes of MoAs. The study can, thereby, present a starting point to further investigate the measurements' content and validity and inform improvements to the MoA Ontology through its application. The ultimate goal of this work is to create more informed and efficacious interventions to improve health and well‐being.

## MATERIALS AND METHODS

This study was reported according to consolidated criteria for reporting qualitative research (COREQ), where relevant (Tong et al., 2007). The study involved a deductive mapping of measurements guided by the MoA Ontology. Throughout the study, the judgements to refine the measure–MoA links were made by two researchers with doctoral degrees relating to psychology, one working as assistant professor (TC—White, cis‐gender female) and another as research fellow (PS—White, cis‐gender female). The first researcher had experience in applying and organizing measurement related to MoAs in repositories, while the second researcher had experience in developing and applying ontologies.

### Materials

#### Measurements

The 167 measure–MoA links (Cornelius et al., 2023) between the 44 self‐report measures in the SOBC Measures Repository (https://measures.scienceofbehaviorchange.org) and the 26 MoAs identified by the HBCP (Carey et al., 2019; Connell et al., 2019; Johnston et al., 2018) served as the starting point for the present study. In contrast to the original study, based on expert feedback stating that some subscales within a given measure seemed to assess substantially different constructs, measure‐MoA links were separated out by subscale as applicable (see Supplementary Material S1). The starting assumption was made that if a measure was linked to one of the 26 MoAs, then so was its subscale. Thus, instead of 44 measures, there were 131 unique measure and subscale combinations, and the number of links increased from 167 to 529 (e.g., the 10‐Item Personality inventory was linked to 3 MoAs in the expert opinion study—considering these links separately across the five subscales resulted in 15 measure–MoA links). For simplicity, both measures without subscales and measures separated into subscales will be referred to as ‘measure’ in the Methods and Results sections.

#### MoAs

The classes of MoAs from the MoA Ontology part of the Behaviour Change Intervention Ontology were used in this study. As the ontology was not published at the start of the current work, an earlier version of the ontology (published on OSF in November 2022), with 270 classes, was used (see Version 5 here: https://osf.io/pkq4e). The ontology has been reported in a paper with 284 classes (see log of 12 added classes: https://osf.io/6gzuk). Since then, further updates to the ontology have resulted in 621 classes (https://github.com/HumanBehaviourChangeProject/ontologies/tree/master/MechanismOfAction; release date: 18 July 2025). However, such changes are expected, given the evolving nature of ontologies. The use of unique identifiers (IDs) for each ontology class ensures that the version applied in this study remains traceable and consistent with the latest version.

### Procedure

The two researchers first independently judged whether each measure was linked to classes of MoAs from the MoA Ontology, recording their judgements on an Excel spreadsheet. This involved the researchers reading each measure's items and then separately coding which classes best captured these items. More than one class could be coded for each measure. To inform their coding, the researchers used iteratively developed guidelines (see Supplementary Material S2).

Measure–MoA links from the expert opinion study (Cornelius et al., 2023) served as a starting point for these judgements. If a class from the ontology was coded for a measure and this class's definition captured the MoA definition from the expert opinion study, then the newly coded measure‐MoA link was considered to be ‘confirmed’. For instance, the measure ‘Future Orientation Scale’ was linked to the MoAs ‘Memory, Attention and Decision Processes’ in Cornelius et al. (2023). Because a related class from the ontology, ‘decision‐making’, mapped on to this measure in the present study, this was considered ‘confirmed’. An original measure–MoA link could be ‘confirmed’ by one or more classes (e.g., a link to the original MoA of self‐regulation could be confirmed by links to both ‘self‐regulation process’ and ‘self‐regulation capability’). Conversely, if the previously coded MoA was not captured by a class from the ontology, the link determined by expert opinion was considered ‘removed’. Classes that were coded and did not correspond to previously coded MoAs were ‘new’ links.

To draft the coding guidelines, the researchers first independently coded 15 measures (three scales with altogether 15 subscales) as a pilot and discussed and reconciled their disagreements. Given the diversity of the remaining measures (e.g., cognitive reasoning and environmental measures), they then independently coded 3–5 measurement scales at a time, discussed and reconciled disagreements and iteratively updated the guidelines. Once the coding process was complete, they jointly reviewed and discussed the final decisions for all measures to ensure that the guidelines had been applied consistently.

### Data analysis strategy

We calculated percent agreement for all researcher judgments on measure‐MoA links made during the initial round of coding. We additionally calculated the number of codes that were changed during final review, after the iteratively developed codebook was complete and quality checks were done to ensure consistency.

Frequency of ‘confirmed’ and ‘removed’ links from the expert opinion study were computed; the frequency of MoA classes from the ontology for both ‘confirmed’ and ‘new’ links are presented in graphical format.

To highlight correspondence between the 26 original MoAs and classes of MoAs from the ontology, a mapping of the previously coded MoA to the ontology's classes for ‘confirmed’ links is presented. Summary statistics for the number of classes for each measure are also reported.

Although each class in the MoA Ontology is considered to be distinct, it is probable that these classes are not assessed separately by the measures (i.e., the measure is not a ‘pure’ measure of a given class). Thus, we identified commonly co‐occurring classes within a given measure (i.e., classes showed up in combination at least five times) for groups of two or more classes.

Finally, notes taken during the coding process were reviewed after reconciliation was complete to compile a list of classes potentially missing from the MoA Ontology. These suggested classes were submitted to the ontology developers for consideration in future iterations of the MoA Ontology.

## RESULTS

The percent agreement between the two researchers' initial coding of classes of MoAs onto measures was 59.4% (see the researchers' coding record here: https://osf.io/pd9fc). Key disagreements related to the complexity of deciding which specific classes should be included to capture measurement items, particularly regarding (1) whether an item actually captures an MoA (e.g., whether a measure of self‐regulating emotion not only captures the self‐regulation process itself but the emotion being regulated) and (2) the granularity of the classes coded (e.g., ‘self‐efficacy belief for a behaviour’ vs. ‘self‐efficacy belief for a behaviour and its outcome’). Following reconciliations, 1122 judgements were recorded as links (678 judgements) or non‐links (444 judgements). During the final review, 71 judgements were updated when the researchers jointly reviewed their reconciled results (93.7% agreement between judgements in the initial reconciliation and review), and 55 additional links were recorded.

Of the 167 original links from the expert opinion study, 108 were considered to be confirmed (64.67%; i.e., the measure or one of its subscales corresponded to a class from the ontology that was captured by the original MoA), and 59 were removed (35.33%). These are summarized in Table 2.

**TABLE 2:** Confirmed and removed links from the original expert opinion study.

Measure	Original MoA	Link result
10‐Item Personality Inventory (TIPI)	Emotion	✓
TIPI	Self‐Image	✓
TIPI	Social/Professional Role & Identity	✗
Behavioural Inhibition and Activation Systems (BIS‐BAS) Scales	Behavioural Regulation	✓
BIS/BAS	Goals	✓
BIS/BAS	Motivation	✓
BIS/BAS	Reinforcement	✓
BIS/BAS	Self‐Image	✓
Barratt Impulsiveness Scale (BIS)	Behavioural Regulation	✓
BIS	Beliefs About Capabilities	✗
BIS	Emotion	✗
BIS	Memory, Attention and Decision Processes	✓
BIS	Self‐Image	✓
Behaviour Rating Inventory of Executive Function (BRIEF)—Adults	Behavioural Regulation	✓
BRIEF—Adults	Beliefs About Capabilities	✓
BRIEF—Adults	Memory, Attention and Decision Processes	✓
BRIEF—Adults	Self‐Image	✗
BRIEF—Adults	Skills	✗
Brief COPE	Behavioural Regulation	✓
Brief COPE	Beliefs About Capabilities	✗
Brief COPE	Emotion	✓
Brief COPE	Self‐Image	✗
Brief COPE	Skills	✓
Brief Risk‐Resilience Index for Screening (BRISC)	Behavioural Regulation	✓
BRISC	Beliefs About Capabilities	✓
BRISC	Emotion	✓
BRISC	Self‐Image	✓
BRISC	Skills	✗
Brief Self‐Control (BSC)	Behavioural Regulation	✓
BSC	Beliefs About Capabilities	✓
BSC	Self‐Image	✓
BSC	Skills	✗
Cognitive Reflection Test (CRT)	Behavioural Regulation	✓
CRT	Memory, Attention and Decision Processes	✓
CRT	Skills	✗
Consideration of Future Consequences (CFC) Scale	Behavioural Regulation	✓
CFC Scale	Beliefs About Consequences	✗
CFC Scale	Goals	✓
CFC Scale	Memory, Attention and Decision Processes	✓
CFC Scale	Motivation	✓
CFC Scale	Values	✓
Couple Coercion Scale	Behavioural Regulation	✓
Couple Coercion Scale	Emotion	✓
Couple Coercion Scale	Social Influences	✓
Daily Inventory of Stressful Events (DISE)	Emotion	✓
DISE	Environmental Context and Resources	✓
DISE	Perceived Susceptibility /Vulnerability	✓
Deferment of Gratification Scale	Behavioural Regulation	✓
Deferment of Gratification Scale	Memory, Attention and Decision Processes	✗
Deferment of Gratification Scale	Motivation	✗
Deferment of Gratification Scale	Reinforcement	✗
Dickman Functional and Dysfunctional Impulsivity Inventory (DII)	Behavioural Regulation	✓
DII	Beliefs About Capabilities	✓
DII	Memory, Attention and Decision Processes	✓
DII	Self‐Image	✗
Domain Specific Risk‐Taking Survey (DOSPERT)—Expected Benefits	Attitudes Towards Behaviour	✗
DOSPERT—Expected Benefits	Behavioural Regulation	✗
DOSPERT—Expected Benefits	Beliefs About Consequences	✓
DOSPERT—Expected Benefits	Memory, Attention and Decision Processes	✗
DOSPERT—Risk Perceptions	Attitudes Towards Behaviour	✗
DOSPERT—Risk Perceptions	Beliefs About Consequences	✓
DOSPERT—Risk Perceptions	Memory, Attention and Decision Processes	✗
DOSPERT—Risk Perceptions	Perceived Susceptibility /Vulnerability	✓
DOSPERT—Risk Taking	Attitudes Towards Behaviour	✗
DOSPERT—Risk Taking	Beliefs About Consequences	✗
DOSPERT—Risk Taking	Intentions	✓
DOSPERT—Risk Taking	Perceived Susceptibility /Vulnerability	✗
DOSPERT—Risk Taking	Self‐Image	✗
Ecological Momentary Assessment of Stressful Events	Emotion	✓
Ecological Momentary Assessment of Stressful Events	Environmental Context and Resources	✓
Emotion Regulation Questionnaire (ERQ)	Behavioural Regulation	✓
ERQ	Beliefs About Capabilities	✗
ERQ	Emotion	✓
ERQ	Memory, Attention and Decision Processes	✗
ERQ	Skills	✗
Emotion Regulation Strategies Scale	Behavioural Regulation	✓
Emotion Regulation Strategies Scale	Emotion	✓
Emotion Regulation Strategies Scale	Skills	✗
Five Facets of Mindfulness Questionnaire (FFMQ)	Behavioural Regulation	✓
FFMQ	Emotion	✓
FFMQ	Memory, Attention and Decision Processes	✓
FFMQ	Self‐Image	✗
FFMQ	Skills	✗
Future Orientation Scale	Behavioural Regulation	✓
Future Orientation Scale	Goals	✗
Future Orientation Scale	Memory, Attention and Decision Processes	✓
Future Orientation Scale	Skills	✗
Future Time Perspective Scale (FTP)	General Attitudes/Beliefs	✓
FTP	Optimism	✗
Generalized Self‐Efficacy Scale (GSE)	Behavioural Regulation	✓
GSE	Beliefs About Capabilities	✓
GSE	Optimism	✗
GSE	Self‐Image	✗
GSE	Skills	✗
Grit‐S	Behavioural Regulation	✓
Grit‐S	Beliefs About Capabilities	✗
Grit‐S	Goals	✓
Grit‐S	Motivation	✗
Grit‐S	Self‐Image	✓
I‐7: Impulsiveness and Venturesomeness Questionnaire	Behavioural Regulation	✓
I‐7: Impulsiveness and Venturesomeness Questionnaire	Emotion	✓
I‐7: Impulsiveness and Venturesomeness Questionnaire	Memory, Attention and Decision Processes	✓
I‐7: Impulsiveness and Venturesomeness Questionnaire	Self‐Image	✓
Kessler Psychological Distress Scale (K6+)	Emotion	✓
K6+	Self‐Image	✓
Mindful Attention Awareness Scale (MAAS)	Behavioural Regulation	✓
MAAS	Memory, Attention and Decision Processes	✓
MAAS	Skills	✗
Multidimensional Assessment of Interoceptive Awareness (MAIA)	Behavioural Regulation	✓
MAIA	Emotion	✓
MAIA	Memory, Attention and Decision Processes	✓
MAIA	Self‐Image	✗
MAIA	Skills	✗
Multidimensional Personality Questionnaire (MPQ): Control vs. Impulsivity Scale	Behavioural Regulation	✓
MPQ: Control vs. Impulsivity Scale	Memory, Attention and Decision Processes	✗
MPQ: Control vs. Impulsivity Scale	Self‐Image	✓
NIH Self‐Efficacy Scale	Behavioural Regulation	✓
NIH Self‐Efficacy Scale	Beliefs About Capabilities	✓
NIH Self‐Efficacy Scale	Optimism	✗
NIH Self‐Efficacy Scale	Self‐Image	✗
NIH Self‐Efficacy Scale	Skills	✗
Parent Cognition Scale	Social Influences	✓
Parent–Child Coercion Scale	Social Influences	✓
Parent–Child Coercion Scale	Social/Professional Role and Identity	✗
Parent‐rated Stress (NIH Perceived Stress Scale)	Behavioural Regulation	✓
Parent‐rated Stress (NIH Perceived Stress Scale)	Emotion	✓
Pearlin Mastery Scale	Behavioural Regulation	✗
Pearlin Mastery Scale	Beliefs About Capabilities	✓
Pearlin Mastery Scale	Optimism	✗
Pearlin Mastery Scale	Self‐Image	✗
Positive and Negative Affect Scheduled (PANAS)	Emotion	✓
PANAS—Child	Emotion	✓
SIDES Affect Dysregulation Scale (Child‐Reported)	Behavioural Regulation	✓
SIDES Affect Dysregulation Scale (Child‐Reported)	Beliefs About Capabilities	✗
SIDES Affect Dysregulation Scale (Child‐Reported)	Emotion	✓
SIDES Affect Dysregulation Scale (Child‐Reported)	Self‐Image	✗
SIDES Affect Dysregulation Scale (Child‐Reported)	Skills	✗
Selection, Optimization, and Compensation (SOC) Questionnaire	Behavioural Regulation	✓
SOC Questionnaire	Beliefs About Consequences	✗
SOC Questionnaire	Goals	✓
SOC Questionnaire	Memory, Attention and Decision Processes	✓
SOC Questionnaire	Self‐Image	✗
Short Self‐Regulation Questionnaire	Behavioural Regulation	✓
Short Self‐Regulation Questionnaire	Beliefs About Capabilities	✓
Short Self‐Regulation Questionnaire	Goals	✓
Short Self‐Regulation Questionnaire	Self‐Image	✓
Short Self‐Regulation Questionnaire	Skills	✓
Theories of Willpower Scale	Behavioural Regulation	✗
Theories of Willpower Scale	Beliefs About Capabilities	✓
Theories of Willpower Scale	Beliefs About Consequences	✓
Theories of Willpower Scale	General Attitudes/Beliefs	✓
Three Factor Eating Questionnaire R‐18 (TFEQ‐r18)	Behavioural Cueing	✓
TFEQ‐r18	Behavioural Regulation	✓
TFEQ‐r18	Beliefs About Capabilities	✓
TFEQ‐r18	Beliefs About Consequences	✓
TFEQ‐r18	Emotion	✓
TFEQ‐r18	Self‐Image	✗
UPPS‐P Impulsive Behaviour Scale	Behavioural Regulation	✓
UPPS‐P	Beliefs About Capabilities	✓
UPPS‐P	Memory, Attention and Decision Processes	✓
UPPS‐P	Self‐Image	✓
Zimbardo Time Perspective Inventory (ZTPI)	Behavioural Regulation	✓
ZTPI	Beliefs About Consequences	✓
ZTPI	Optimism	✗
ZTPI	Self‐Image	✗
Zuckerman Sensation Seeking Survey‐V	Behavioural Regulation	✓
Zuckerman Sensation Seeking Survey‐V	Self‐Image	✗

**FIGURE 2:**
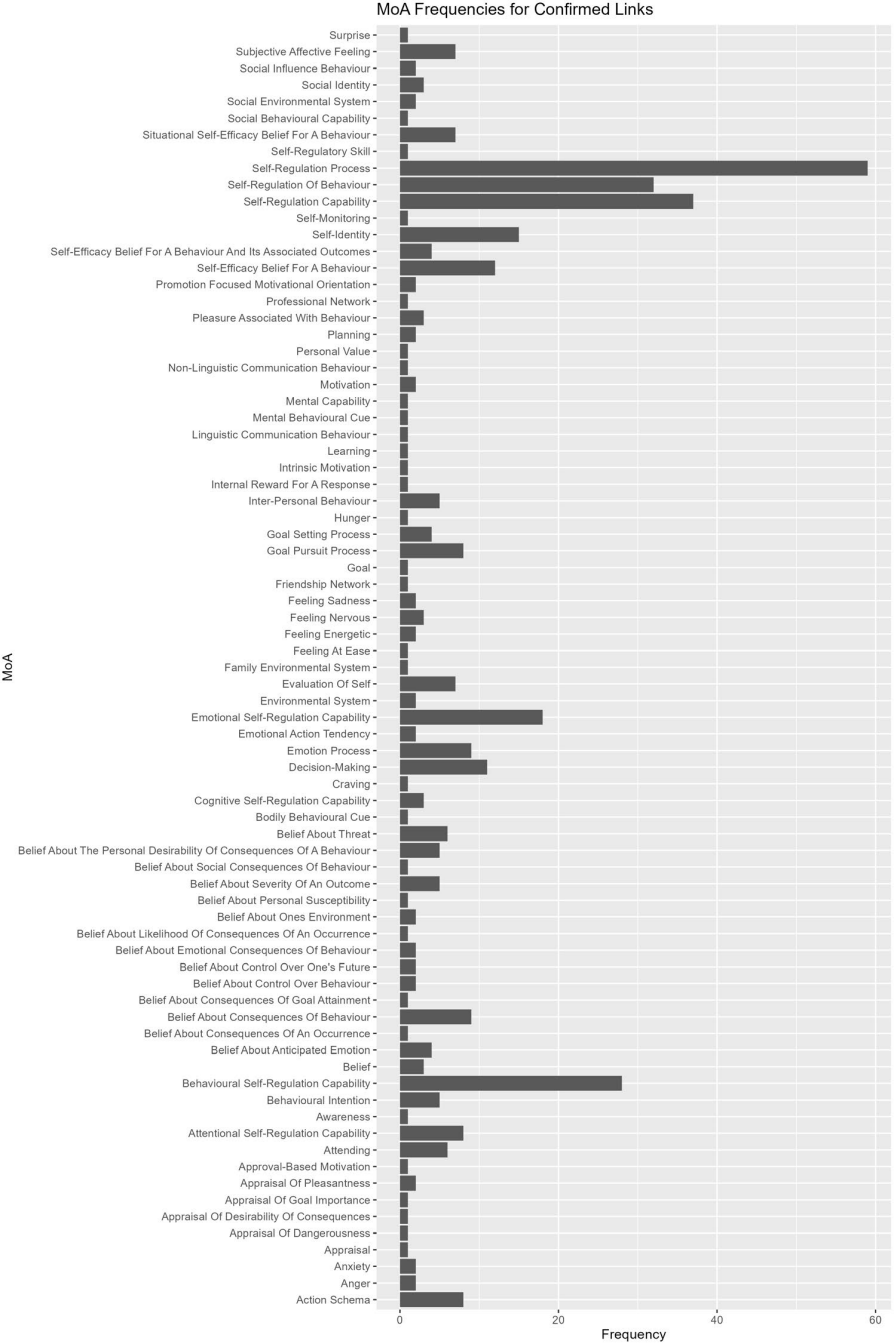
Frequency of new classes from the Mechanism of Action (MoA) Ontology that confirmed measure–MoA links from the earlier study.

**FIGURE 3:**
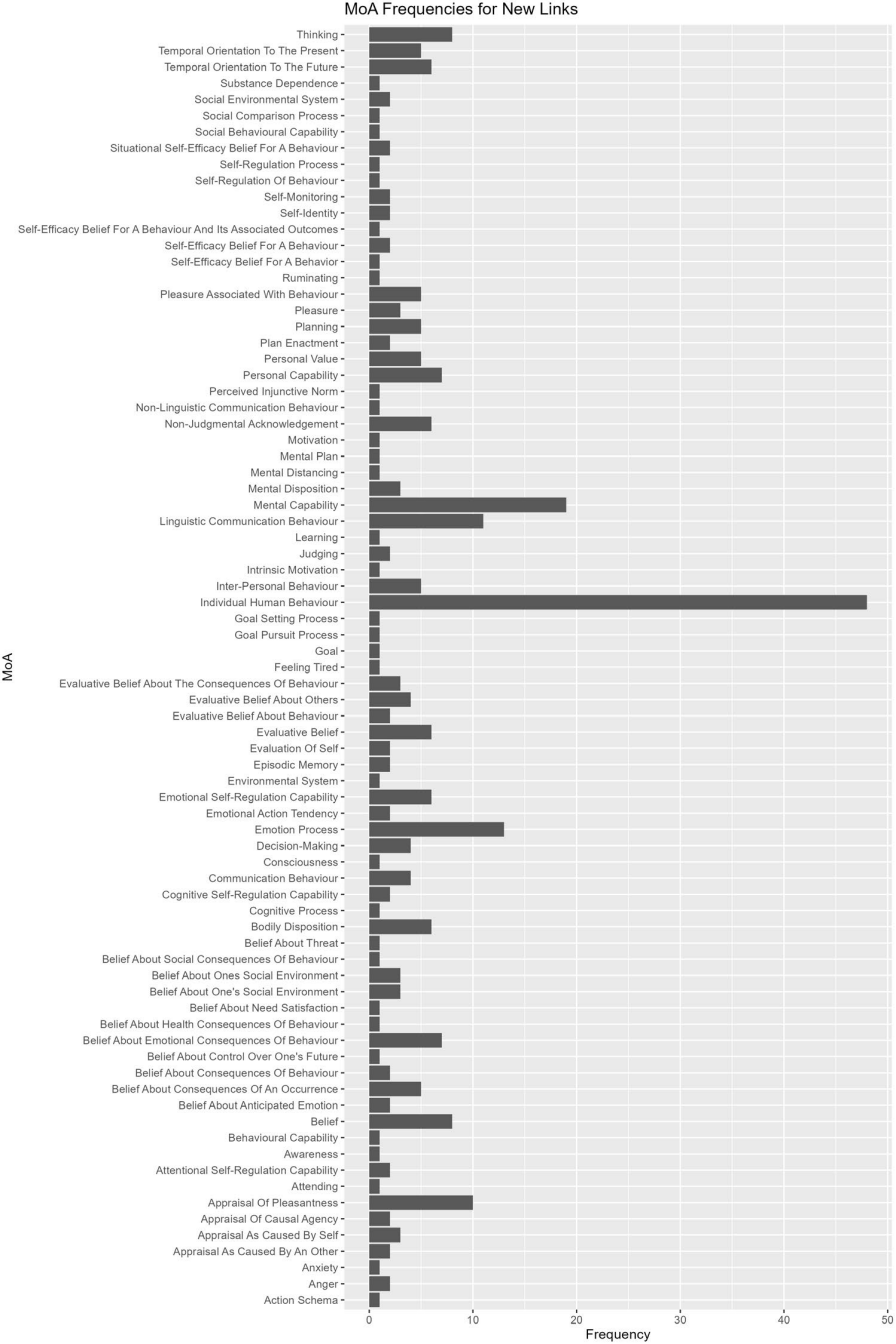
Frequency of new classes from the Mechanism of Action (MoA) Ontology that were part of new links.

Within the new ontology, and separated by subscale, there were a total of 397 links that confirmed expert opinion, 289 new links, and 309 removed links (https://osf.io/pd9fc). Figures 2 and 3 depict the frequency of the new MoAs when these were confirmed (Figure 2) or new (Figure 3).

There were 5.24 MoAs on average for the 131 measure/subscale combinations (SD = 3.78; Median = 5.0; Range 1.0, 28.0). The mapping of old MoAs to the MoAs from the ontology is in Supplementary Material S3. For example, for measurements relevant to the original MoA ‘Goals’, the new mapping included five classes from the MoA Ontology: ‘appraisal of goal importance’ (MFOEM:000072), ‘belief about consequences of goal attainment’ (BCIO:006021), ‘goal’ (BCIO:006049), ‘goal pursuit process’ (BCIO:006096) and ‘goal setting process’ (BCIO:006114).

To see whether certain MoAs in the ontology tended to be coded together (i.e., were often coded together within a single Measure/Subscale combination), we looked to see which MoAs were coded in groups of 2 through 10 at least 5 or more times. All common combinations are in Supplementary Material S4. No combinations of 7 or more MoAs were coded at least 5 times across each measure/subscale. The most common pairing, reoccurring for 36 measures, was ‘self‐regulation capability’ (BCIO:006005) and ‘self‐regulation process’ (BCIO:050268).

A total of 23 classes (e.g., self‐efficacy belief for mental process) were identified as potentially missing from the MoA Ontology and suggested labels and definitions for these classes (see the full list of classes here: https://osf.io/6gzuk). This list has been shared with the MoA Ontology development team for consideration. The need for greater specification of classes about self‐regulatory processes also became apparent throughout this process because self‐regulation processes involve two parts (i.e., process A to regulate process B). For example, one may use behaviour to regulate an emotional process or, vice versa, emotions may be used by an individual to regulate their behaviour. This issue has also been raised with the MoA Ontology developers.

## DISCUSSION

Building on the previous mapping of 44 self‐report measures from the SOBC repository onto 26 broad MoAs, the current study provided a more granular mapping of the 131 scales in these measures onto 270 clearly defined and distinct classes from an expanded MoA Ontology. This study also served to provide data regarding the distinctness of MoAs in practice (i.e., identify patterns of co‐occurrence) and to identify potentially missing classes from the MoA Ontology. There were a total of 529 links between measures (subscales) and MoAs. Of these, 397 were considered to confirm links from the expert opinion study (Cornelius et al., 2023), and 289 were new. Many classes were commonly coded together for a given measure (subscale), and 23 new classes were suggested for inclusion in future iterations of the MoA Ontology.

Study results provide more detailed guidance for researchers considering how to best measure an MoA hypothesized to underlie behaviour change and should encourage careful attention to correspondence between theory and methodology. MoAs should be clearly defined, distinct and measured both precisely and completely. Separating out measures by subscale and utilizing an expanded set of MoAs enabled greater precision. For example, although the two subscales of the Emotion Regulation Questionnaire, Cognitive Reappraisal (6 items; e.g., ‘When I want to feel a more positive emotion (such as joy or amusement), I change what I am thinking about’) and Expressive Suppression (4 items; e.g., ‘When I am feeling negative emotions, I make sure not to express them’), are related, the processes they measure are distinct (Gross & John, 2003). Furthermore, whereas the expert opinion study had only one broad MoA for ‘Emotion’, classes from the MoA Ontology were separated into more clear and distinct MoAs (e.g., ‘anger’ and ‘happiness’). The greater level of detail in the MoAs specified within the ontology serves as a precise and granular classification framework for describing MoAs in reports and for synthesizing and organizing evidence and measurements. Simpler frameworks, such as the Theoretical Domains Framework (TDF; Cane et al., 2012), have the advantage of being easier to apply, but the disadvantage of being high level and therefore lacking the detail of the MoA Ontology. Reasons for easier application include fewer categories, and definitions that capture a wider range of potential applications. Recent work has mapped the MoA Ontology's expanded classes to the 26 MoAs included in the Theories and Techniques Tool (TaTT) (Zhang et al., 2025) and the previous work to map measurements to MoAs (Cornelius et al., 2023). These 26 MoAs also include the 14 domains of the TDF, thereby providing a method for starting with a broad domain in the TDF (e.g., ‘Emotion’) and then identifying more detailed corresponding subclasses within the MoA Ontology.

Classes within the MoA Ontology are specified such that definitions do not overlap, but rather each class represents varying specificity within a hierarchical structure. Within this organizing framework, classes may commonly co‐occur (i.e., frequently be linked in common groupings to different measures), and this may be due to different reasons. First, it is possible that co‐occurrence indicates child classes within a shared parent class, such that a measure assesses multiple facets of an overarching construct. As an example, the Positive and Negative Affect Scales (PANAS; Watson et al., 1988) included the classes ‘feeling at ease’ (MFOEM:000107) and ‘feeling nervous’ (MFOEM:000124) both of which are children of the parent class ‘subjective affective feeling’ (MFOEM:000006). If all child classes are captured by a measure, this may also indicate that this measure captures the parent class completely. However, such claims should be made with caution because they assume that every possible class has been included within the ontology. Co‐occurrence can also indicate that certain classes may have non‐overlapping definitions but be practically indistinguishable in some measurement contexts. The most commonly co‐occurring pair, ‘self‐regulation process’ (BCIO:050268) and ‘self‐regulation capability’ (BCIO:006005), reflects this tautology (and is also reflected in the coding rules). Specifically, if someone is using a self‐regulation process, they must also be capable of using this process. Note that the converse is not true, as someone may be capable of self‐regulation but not implement these skills. This implies that some MoAs may not be separable using self‐report measures, and certainly merits greater attention to both improve measurement practices and parse what this means for existing theories.

After the coding process, potentially missing classes from the MoA Ontology were identified from meeting notes. The greatest difficulty was in coding MoAs that were implicit. For example, no class accurately described social influences via non‐conscious processes such as emotion contagion—as seen in their labels, both ‘inter‐personal behaviour’ (BCIO:036025) and ‘social influence behaviour’ (BCIO:006099) required a behaviour, defined as ‘A bodily process of a human that involves co‐ordinated contraction of striated muscles controlled by the brain’. It was also challenging to code complex self‐regulatory processes, in which process A regulates process B (e.g., reframing one's thoughts to regulate smoking behaviour vs. changing one's behaviours as a method of influencing thoughts). Greater specificity in entities related to self‐regulation could support more precise coding. A key advantage of the MoA Ontology is that it can be updated based on evidence and feedback (Arp et al., 2015; He et al., 2018), with new classes being added where needed, and with historical data allowing prior coding to be included in updated version due to backwards compatibility. For instance, since the mapping work reported here, the ontology has been updated to include ‘affective attitudes’ to more fully capture attitudes proposed within theories (Schenk et al., 2024). With the 23 classes proposed as a result of the mapping work reported here, this study supports expanding the applicability of the ontology to wider use cases.

## LIMITATIONS AND FUTURE WORK

Results are limited by the inclusion of only the 44 self‐report measures included in the SOBC Measures Repository, which is a non‐comprehensive set of selected measures, so findings may not generalize. Furthermore, these measures were developed without reference to the MoA Ontology, so lack of correspondence can be expected. That said, this study was intended to be illustrative (rather than comprehensive), many of these measures are widely used (showing the breadth of the issue), and the insights gained from this effort can be used to inform the creation of new measures and refinement of existing ones to improve rigour and transparency and to inform theories of behaviour change. Insight into the unique challenges of cumulating the existing literature (e.g., through meta‐analysis) due to measure heterogeneity and overlapping constructs was also gained. Due to this complexity, other methodological approaches have advocated for developing new measures for specific studies, so long as constructs are clearly labelled and defined (Peters & Crutzen, 2022). Underspecified or ambiguous measure items (e.g., ‘I am constantly without money’) also contributed to difficulty in linking measures (subscales) to classes from the MoA Ontology and should raise concern regarding the clarity and quality of these measures. Some measure items referenced more stable influences on behaviour (e.g., structural environment, physical health), which would be better captured by the Setting or Population Ontologies part of the Behaviour Change Intervention Ontology (Michie et al., 2017; Norris et al., 2020). Finally, updates to the MoA Ontology are ongoing, and we used an earlier version of the ontology that can be referenced on the Open Science Framework (OSF; https://osf.io/pkq4e). However, the use of unique IDs for each class in the ontology also means that any changes (e.g., their labels) to these classes can be tracked.

The judgements made during coding were based on two researchers' knowledge and subjective interpretations of the measurement items and the ontology classes. Both researchers have led work on measure classification and ontology development, including the measures and MoAs examined in the current study. Despite this and the application of a standardized framework, personal biases may have unintentionally impacted study results and generalizability. This study is an extension to prior work using similar methodologies (e.g., mapping measures to a smaller set of MoAs, but via contribution from a larger group of experts; Cornelius et al., 2023). To illustrate this programmatic approach, the current study includes a transparent comparison in the two mappings produced between MoAs and their potential measurements. The mapping generated in the current study should be viewed as a detailed starting point for linking measures to relevant MoAs. Quantitative studies are underway that map items to mechanisms with larger sample sizes, for example, using discriminant content validity. These heterogeneous methodologies allow for a nuanced and comprehensive exploration of the complex issues surrounding measurement of MoAs. This work should continue to be expanded across a broad range of measures, with potential to improve rigour, transparency, theory development and intervention efficacy and potentially even provide more formal guidance on measure selection in behavioural process research.

## CONCLUSION

The present study builds on previous research by providing more precise guidance for behavioural scientists when selecting measures of MoAs for use in research studies. Results also highlight the critical importance of operationalizing behavioural MoAs to ensure rigorous and valid hypothesis testing. For example, the finding that different measures often tapped into multiple MoAs, as well as the common co‐occurrence of MoAs, suggests challenges in measuring certain MoAs in isolation. Future efforts should continue to tackle the development and refinement of measures that are valid for assessing behavioural MoAs. Insights were also generated regarding missing classes, which can be used as feedback to inform future iterations of the MoA Ontology.

## AUTHOR CONTRIBUTIONS

**Paulina M. Schenk:** Conceptualization; data curation; formal analysis; investigation; methodology; project administration; validation; writing – original draft; writing – review and editing; visualization. **Susan Michie:** Supervision; writing – review and editing; funding acquisition. **Marie Johnston:** Funding acquisition; writing – review and editing; supervision. **Talea Cornelius:** Funding acquisition; conceptualization; data curation; formal analysis; investigation; methodology; project administration; software; supervision; validation; writing – original draft; writing – review and editing; visualization.

## CONFLICT OF INTEREST STATEMENT

The authors declare that they have no conflicts of interest.

## Supporting information


Data S1:

## Data Availability

There is no registration or pre‐registration plan available for the study or analysis. All data, code and materials are available at https://osf.io/mt6vw/.
Data set: https://osf.io/qt5ay/?view_only=9e48ae0353e04de99df0859e16e6f214 (*link view only for masked review*). R code: https://osf.io/ebazx/?view_only=9e48ae0353e04de99df0859e16e6f214 (*link view only for masked review*). Coding rules: https://osf.io/xdgy9/?view_only=9e48ae0353e04de99df0859e16e6f214 (*link view only for masked review*). Measurement items and judgements on their mapping to the MoA Ontology for each researcher: https://osf.io/pd9fc/?view_only=9e48ae0353e04de99df0859e16e6f214 (*link view only for masked review*). Suggested changes to the MoA Ontology: https://osf.io/6gzuk/?view_only=9e48ae0353e04de99df0859e16e6f214 (*link view only for masked review*).

## References

[bjhp70015-bib-0001] Arp, R. , Smith, B. , & Spear, A. D. (2015). Building ontologies with basic formal ontology. Mit Press.

[bjhp70015-bib-0002] Bohlen, L. C. , Carey, R. , de Bruin, M. , Rothman, A. , Johnston, M. , Kelly, M. P. , & Michie, S. (2019). Links between behaviour change techniques and mechanisms of action: An expert consensus study. Annals of Behavioral Medicine, 53(8), 708–720. 30452535 10.1093/abm/kay082 PMC6636885

[bjhp70015-bib-0003] Cane, J. , O'Connor, D. , & Michie, S. (2012). Validation of the theoretical domains framework for use in behaviour change and implementation research. Implementation Science, 7, 1–17. 10.1186/1748-5908-7-37 PMC3483008 22530986

[bjhp70015-bib-0004] Carey, R. N. , Connell, L. E. , Johnston, M. , Rothman, A. J. , De Bruin, M. , Kelly, M. P. , & Michie, S. (2019). Behavior change techniques and their mechanisms of action: A synthesis of links described in published intervention literature. Annals of Behavioral Medicine, 53(8), 693–707. 30304386 10.1093/abm/kay078 PMC6636886

[bjhp70015-bib-0005] Connell, L. E. , Carey, R. N. , De Bruin, M. , Rothman, A. J. , Johnston, M. , Kelly, M. P. , & Michie, S. (2019). Links between behavior change techniques and mechanisms of action: An expert consensus study. Annals of Behavioral Medicine, 53(8), 708–720. 30452535 10.1093/abm/kay082 PMC6636885

[bjhp70015-bib-0006] Cornelius, T. , Derby, L. , Connell Bohlen, L. , Birk, J. L. , Rothman, A. J. , Johnston, M. , & Michie, S. (2023). Linking measures to mechanisms of action: An expert opinion study. British Journal of Health Psychology, 28(1), 98–115. 35781731 10.1111/bjhp.12614 PMC9807686

[bjhp70015-bib-0007] Derby, L. , Bohlen, L. C. , Michie, S. , Johnston, M. , Birk, J. L. , Rothman, A. J. , & Cornelius, T. (2024). Linking measures to mechanisms of action in behavior change: A qualitative analysis of expert views. Social Science & Medicine, 352, 117023. 38820694 10.1016/j.socscimed.2024.117023 PMC11472330

[bjhp70015-bib-0008] Edmondson, D. , Falzon, L. , Sundquist, K. J. , Julian, J. , Meli, L. , Sumner, J. A. , & Kronish, I. M. (2018). A systematic review of the inclusion of mechanisms of action in NIH‐funded intervention trials to improve medication adherence. Behaviour Research and Therapy, 101, 12–19. 29033097 10.1016/j.brat.2017.10.001 PMC5800992

[bjhp70015-bib-0009] Gross, J. J. , & John, O. P. (2003). Individual differences in two emotion regulation processes: Implications for affect, relationships, and well‐being. Journal of Personality and Social Psychology, 85(2), 348–362. 12916575 10.1037/0022-3514.85.2.348

[bjhp70015-bib-0010] Gupta, V. (2023). What is repository? (Definition, Tutorial, How to Clone). Built In. https://builtin.com/software‐engineering‐perspectives/repository

[bjhp70015-bib-0011] Hastings, J. (2017). Primer on ontologies. In C. Dessimoz & N. Škunca (Eds.), The Gene Ontology Handbook. Springer New York, 3–13.

[bjhp70015-bib-0012] He, Y. , Xiang, Z. , Zheng, J. , Lin, Y. , Overton, J. A. , & Ong, E. (2018). The eXtensible ontology development (XOD) principles and tool implementation to support ontology interoperability. Journal of Biomedical Semantics, 9, 1–10. 29329592 10.1186/s13326-017-0169-2 PMC5765662

[bjhp70015-bib-0013] Johnston, M. , Carey, R. N. , Connell Bohlen, L. , Johnston, D. W. , Rothman, A. , de Bruin, M. , & Michie, S. (2018). Linking behavior change techniques and mechanisms of action: Triangulation of findings from literature synthesis and expert consensus. Annals of Behavioral Medicine, 53(8), 708–720. 10.1093/abm/kay082 PMC6636885 30452535

[bjhp70015-bib-0014] Johnston, M. , Carey, R. N. , Connell Bohlen, L. E. , Johnston, D. W. , Rothman, A. J. , De Bruin, M. , Kelly, M. P. , Groarke, H. , & Michie, S. (2021). Development of an online tool for linking behavior change techniques and mechanisms of action based on triangulation of findings from literature synthesis and expert consensus. Translational Behavioral Medicine, 11(5), 1049–1065. 32749460 10.1093/tbm/ibaa050 PMC8158171

[bjhp70015-bib-0015] Michie, S. , Thomas, J. , Johnston, M. , Aonghusa, P. M. , Shawe‐Taylor, J. , Kelly, M. P. , Deleris, L. A. , Finnerty, A. N. , Marques, M. M. , & Norris, E. (2017). The human behaviour‐change project: Harnessing the power of artificial intelligence and machine learning for evidence synthesis and interpretation. Implementation Science, 12(1), 1–12. 29047393 10.1186/s13012-017-0641-5 PMC5648456

[bjhp70015-bib-0016] Michie, S. , West, R. , Finnerty, A. N. , Norris, E. , Wright, A. J. , Marques, M. M. , Johnston, M. , Kelly, M. P. , Thomas, J. , & Hastings, J. (2020). Representation of behaviour change interventions and their evaluation: Development of the upper level of the behaviour change intervention ontology. Wellcome Open Research, 5, 123. 33614976 10.12688/wellcomeopenres.15902.1 PMC7868854

[bjhp70015-bib-0017] Michie, S. , West, R. , & Hastings, J. (2019). Creating ontological definitions for use in science. Qeios.

[bjhp70015-bib-0018] Norris, E. , Marques, M. M. , Finnerty, A. N. , Wright, A. J. , West, R. , Hastings, J. , Williams, P. , Carey, R. N. , Kelly, M. P. , & Johnston, M. (2020). Development of an intervention setting ontology for behaviour change: Specifying where interventions take place. Wellcome Open Research, 5, 124. 32964137 10.12688/wellcomeopenres.15904.1 PMC7489274

[bjhp70015-bib-0019] Peters, G.‐J. , & Crutzen, R. (2022). Knowing what we're talking about: facilitating decentralized, unequivocal publication of and reference to psychological construct definitions and instructions.

[bjhp70015-bib-0020] Riddle, M. (2015). Science of behavior change working group news from the NIH: Using an experimental medicine approach to facilitate translational research. Translational Behavioral Medicine, 5(4), 486–488. 26622921 10.1007/s13142-015-0333-0 PMC4656226

[bjhp70015-bib-0021] Schenk, P. M. , Wright, A. J. , West, R. , Hastings, J. , Lorencatto, F. , Moore, C. , Hayes, E. , Schneider, V. , & Michie, S. (2024). An ontology of mechanisms of action in behaviour change interventions. Wellcome Open Research, 8, 337. 38481854 10.12688/wellcomeopenres.19489.2 PMC10933577

[bjhp70015-bib-0023] Seppälä, S. , Ruttenberg, A. , & Smith, B. (2017). Guidelines for writing definitions in ontologies. Ciência da Informação, 46(1), 73–88.

[bjhp70015-bib-0024] Sumner, J. A. , Birk, J. L. , Cornelius, T. , Derby, L. , Edmondson, D. , & Davidson, K. W. (2019). The NIH science of behavior change mechanism‐focused approach to behavior change research. Psychosomatic Medicine, 81(4), A178.

[bjhp70015-bib-0025] Sumner, J. A. , Carey, R. N. , Michie, S. , Johnston, M. , Edmondson, D. , & Davidson, K. W. (2018). Using rigorous methods to advance behaviour change science. Nature Human Behaviour, 2(11), 797–799. 10.1038/s41562-018-0471-8 PMC6437667 30931398

[bjhp70015-bib-0026] Tong, A. , Sainsbury, P. , & Craig, J. (2007). Consolidated criteria for reporting qualitative research (COREQ): A 32‐item checklist for interviews and focus groups. International Journal for Quality in Health Care, 19(6), 349–357. 17872937 10.1093/intqhc/mzm042

[bjhp70015-bib-0027] Watson, D. , Clark, L. A. , & Tellegen, A. (1988). Development and validation of brief measures of positive and negative affect: The PANAS scales. Journal of Personality and Social Psychology, 54(6), 1063–1070. 3397865 10.1037//0022-3514.54.6.1063

[bjhp70015-bib-0028] Wright, A. J. , Norris, E. , Finnerty, A. N. , Marques, M. M. , Johnston, M. , Kelly, M. P. , Hastings, J. , West, R. , & Michie, S. (2020). Ontologies relevant to behaviour change interventions: A method for their development. Wellcome Open Research, 5, 126. 33447665 10.12688/wellcomeopenres.15908.1 PMC7786424

[bjhp70015-bib-0029] Zhang, L. , Schenk, P. M. , Santilli, M. , Wright, A. J. , Marques, M. M. , Johnston, M. , & Michie, S. (2025). Linking behaviour change techniques to mechanisms of action: Using the theory and techniques tool alongside the behaviour change intervention ontology: [version 1; peer review: 1 approved, 1 not approved]. Wellcome Open Research, 10, 192. 10.12688/wellcomeopenres.23879.2 PMC12596556 41216361

