# Supplementary material for: Measuring processes of change in behavioural interventions: Insights gained from linking mechanisms of action to associated measures
Source: Br J Health Psychol. 2025 Aug 18;30(3):e70015. doi: 10.1111/bjhp.70015 (PMC12361588; doi:10.1111/bjhp.70015)
Supplement: Supplementary file 1 — Data S1: [file BJHP-30-0-s001.docx]

**Supplementary files**

Supplementary file 1

Measure names and number of subscales for the 44 self-report measures included in the study

| **Scale** | **Number of Subscales** |
| --- | --- |
| Brief COPE | 14 |
| Multidimensional Assessment of Interoceptive Awareness (MAIA) | 8 |
| Barratt Impulsiveness Scale (BIS) | 6 |
| Emotion Regulation Strategies Scale | 6 |
| 10-Item Personality Inventory (TIPI) | 5 |
| Domain Specific Risk-Taking Survey (DOSPERT) - Expected Benefits | 5 |
| DOSPERT - Risk Perceptions | 5 |
| DOSPERT - Risk Taking | 5 |
| Five Facets of Mindfulness Questionnaire (FFMQ) | 5 |
| UPPS-P Impulsive Behavior Scale | 5 |
| Zimbardo Time Perspective Inventory (ZTPI) | 5 |
| Behavior Rating Inventory of Executive Function (BRIEF) - Adults | 4 |
| Behavioral Inhibition and Activation Systems (BIS-BAS) Scales | 4 |
| Daily Inventory of Stressful Events (DISE) | 4 |
| Selection, Optimization, and Compensation (SOC) Questionnaire | 4 |
| Zuckerman Sensation Seeking Survey-V | 4 |
| Brief Risk-Resilience Index for SCreening (BRISC) | 3 |
| I-7: Impulsiveness and Venturesomeness Questionnaire | 3 |
| Three Factor Eating Questionnaire R-18 (TFEQ-r18) | 3 |
| Dickman Functional and Dysfunctional Impulsivity Inventory (DII) | 2 |
| Emotion Regulation Questionnaire (ERQ) | 2 |
| Parent Cognition Scale | 2 |
| Positive and Negative Affect Scheduled (PANAS) | 2 |
| PANAS - Child | 2 |
| Theories of Willpower Scale | 2 |
| Brief Self-Control (BSC) | 0 |
| Cognitive Reflection Test (CRT) | 0 |
| Consideration of Future Consequences (CFC) Scale | 0 |
| Couple Coercion Scale | 0 |
| Deferment of Gratification Scale | 0 |
| Ecological Momentary Assessment of Stressful Events | 0 |
| Future Orientation Scale | 0 |
| Future Time Perspective Scale (FTP) | 0 |
| Generalized Self-Efficacy Scale (GSE) | 0 |
| Grit-S | 0 |
| Kessler Psychological Distress Scale (K6+) | 0 |
| Mindful Attention Awareness Scale (MAAS) | 0 |
| Multidimensional Personality Questionnaire (MPQ): Control vs. Impulsivity Scale | 0 |
| NIH Self-Efficacy Scale | 0 |
| Parent-Child Coercion Scale | 0 |
| Parent-rated Stress (NIH Perceived Stress Scale) | 0 |
| Pearlin Mastery Scale | 0 |
| Short Self-Regulation Questionnaire | 0 |
| SIDES Affect Dysregulation Scale (Child-Reported) | 0 |

Supplementary file 2

**Goal**

Refine the mapping of the SOBC self-report measures to the HBCP MoA Ontology.

- The 167 links established in an earlier study will be used as a starting point to guide this effort; however, new links may be added or removed, and subscales will be examined separately.
- We will:
  - document whether links are confirmed, new, or deleted.
  - use a more specific mapping approach (does the item seem to assess this MoA?)
  - document potentially missing or unclear entities in the ontology.

**Deciding on which class a measurement scale should be mapped onto**

1. For scale/subscale to be mapped onto an entity, at least one measurement item must assess the entity.
   1. If an item assesses an entity that is not specifically captured by the ontology, consider mapping this item onto a more general entity.
   2. Scales/subscales can map onto multiple entities, and items can assess multiple entities.
2. All items within a scale/subscale need to be considered. If a single item is considered to map onto an entity (i.e., the item intends to “measure” this mechanism), code this mechanism.
   1. More specific entities should be coded when possible.
   2. Do NOT code a higher-level entity if all items are captured by a lower-level entity.

**Guidance for specific entities in the MoA Ontology**

**1. Action schema**

- When coding an item as “individual human behaviour,” consider whether this behaviour could follow an activated action schema. If so, also code “action schema”. For example, if a stressor tends to elicit a specific coping response, then this coping response can be considered as guided by an action schema.
- If it is unclear whether the behaviour is self-initiated (e.g., “receiving support”), then this is too underspecified to be coded as “action schema.”

**2. Appraisal**

- Code “appraisal” or its subclasses if someone is evaluating something (e.g., situation, object) and this could have emotional consequences (e.g., was an event upsetting).

**3. Attending/awareness**

- Code “attending” if an item is about a process and “awareness” if an item is about a state.

**4. Belief**

- Code “belief” subclasses (e.g., belief about social environment) only if the item involves conjecture about future states (e.g., based on previous patterns, reactions of others).

**5. Emotion process**

- Code “emotion process” if someone is feeling/experiencing something in the moment (or a potential moment being discussed) *and* if emotion is related to an object or stimulus.

**6. Pleasure/Evaluative belief**

- Code “pleasure” when an item is about something a person likes, but code “belief” if the item is about something they think they *would* like.
- Code “evaluative belief” or its subclasses for items about evaluating something as positive or negative (e.g., “I made mistakes”).

**7. Individual human behaviour**

- Code “individual human behaviour” or its subclasses for items about whether someone does or has done a behaviour.
- Verbal actions (e.g., “making jokes”) are considered behaviour.

**8. Self-identity, evaluation of self and social identity**

- “Self-identity” should be coded when statements query, “I am […]”.
- Code “evaluation of self” only if this has a clear positive or negative connotation (e.g., critical, quarrelsome), but not when this is unclear (e.g., conventional).
- “Social identity” should be coded when talking about oneself relative to others, and not for beliefs about others’ thoughts (e.g., “people would say that I […]”).

**9. Self-regulation process**

- If a person reports that they are self-regulating their thinking and/or behaviour, this implies an ability to self-regulate. Therefore, when coding an item as “self-regulation process” or its subclasses, also consider coding “self-regulation capability” or its relevant subclasses.
- Items that imply the *lack* of a self-regulation process do not provide information about self-regulation capability.
- When someone has self-regulation capabilities, this does not necessarily mean they use a self-regulatory process.

**10. Self-regulation target**

- If someone engages in a behaviour to regulate a non-behavioural target (e.g., emotion), only code the self-regulation target (e.g., “self-regulation of emotion”). However, “individual human behaviour” should be coded, since this item assesses a behaviour that a person engages in.
- If a cognitive or emotional action is intended to regulate something unspecified, code “self-regulation capability” broadly. If the outcome is partially specified, code both the specific and general entities.

**12. Subjective affective feeling**

- Code “subjective affective feeling” if someone reports general feelings (e.g., general sadness).

**13. Capability-related classes**

- Only code “personal capability” for items about general abilities that do not necessarily require self-regulation (e.g., ability to “react quickly”).
- Only code “mental capability” for items about one’s mental ability that do not necessarily require self-regulation. For example, being “good at concentrating” implies attentional self-regulation capabilities, but being “good at math” could involve intuitive capabilities.
  - Code both “self-regulation capability” and “mental capability”, when something requires self-regulation and mental capabilities (e.g., being “good at careful reasoning”, requiring cognitive self-regulation and reasoning capabilities).
- Only code “behavioural capability” for items about a behavioural ability that do not necessarily require self-regulation (e.g., being “good at skiing”).
  - Also consider coding “self-efficacy belief for a behaviour”.

**14. Thinking**

- Code “thinking” for items about thinking processes (e.g., “I think about what to do”), when these cannot be captured by self-regulation (e.g., of emotion: “I try to think about it positively”).

**15. Planning**

- Code “planning” for items about planning processes (e.g., “I plan ahead”), and “mental plan” for items about planning outcomes/lack thereof (e.g., “I buy things I hadn’t planned for”).

Supplementary file 3

Mapping of original MoAs to entities from the MoA Ontology.

| **Original Mechanism** | **New Mechanism with alphanumeric ID** | **n** |
| --- | --- | --- |
| Behavioural Cueing | bodily behavioural cue (BCIO:006092) | 1 |
|  | mental behavioural cue (BCIO:006093) | 1 |
| Behavioural Regulation | action schema (BCIO:006046) | 8 |
|  | behavioural self-regulation capability (BCIO:006006) | 28 |
|  | self-monitoring (BCIO:006137) | 1 |
|  | self-regulation capability (BCIO:006005) | 37 |
|  | self-regulation of behaviour (BCIO:006103) | 32 |
|  | self-regulation process (BCIO:050268) | 59 |
|  | social behavioural capability (BCIO:006011) | 1 |
| Beliefs About Capabilities | belief (MF:0000041) | 1 |
|  | belief about control over one's future (BCIO:006022) | 1 |
|  | self-efficacy belief for a behaviour (BCIO:006154) | 12 |
|  | self-efficacy belief for a behaviour and its associated outcomes (BCIO:006043) | 4 |
|  | situational self-efficacy belief for a behaviour (BCIO:006044) | 7 |
| Beliefs About Consequences | belief about consequences of an occurrence (BCIO:050217) | 1 |
|  | belief about consequences of behaviour (BCIO:006019) | 9 |
|  | belief about emotional consequences of behaviour (BCIO:050218) | 1 |
|  | belief about likelihood of consequences of an occurrence (BCIO:006026) | 1 |
|  | belief about severity of an outcome (BCIO:006030) | 4 |
|  | belief about the personal desirability of consequences of a behaviour (BCIO:006149) | 5 |
| Emotion | anger (MFOEM:000009) | 2 |
|  | anxiety (MFOEM:000028) | 2 |
|  | appraisal of pleasantness (MFOEM:000061) | 1 |
|  | belief about anticipated emotion (BCIO:006140) | 1 |
|  | belief about emotional consequences of behaviour (BCIO:050218) | 1 |
|  | emotion process (MFOEM:000001) | 9 |
|  | emotional action tendency (MFOEM:000007) | 2 |
|  | emotional self-regulation capability (BCIO:006008) | 18 |
|  | feeling at ease (MFOEM:000107) | 1 |
|  | feeling energetic (MFOEM:000109) | 2 |
|  | feeling nervous (MFOEM:000124) | 3 |
|  | feeling sadness (BCIO:050233) | 2 |
|  | hunger (MFOEM:000205) | 1 |
|  | pleasure associated with behaviour (BCIO:006159) | 2 |
|  | subjective affective feeling (MFOEM:000006) | 7 |
|  | surprise (MFOEM:000032) | 1 |
| Environmental Context & Resources | belief about ones environment (BCIO:006029) | 1 |
|  | environmental system (ENVO:01000254) | 2 |
|  | family environmental system (BCIO:006002) | 1 |
|  | friendship network (BCIO:006003) | 1 |
|  | professional network (BCIO:050213) | 1 |
|  | social environmental system (BCIO:006001) | 2 |
| General Attitudes/Beliefs | belief (MF:0000041) | 2 |
|  | belief about control over behaviour (BCIO:006152) | 2 |
|  | belief about control over one's future (BCIO:006022) | 1 |
|  | belief about ones environment (BCIO:006029) | 1 |
| Goals | appraisal of goal importance (MFOEM:000072) | 1 |
|  | belief about consequences of goal attainment (BCIO:006021) | 1 |
|  | goal (BCIO:006049) | 1 |
|  | goal pursuit process (BCIO:006096) | 8 |
|  | goal setting process (BCIO:006114) | 4 |
| Intentions | behavioural intention (BCIO:006016) | 5 |
| Memory, Attention, & Decision Processes | attending (MF:0000018) | 6 |
|  | attentional self-regulation capability (BCIO:050214) | 8 |
|  | awareness (BCIO:006015) | 1 |
|  | cognitive self-regulation capability (BCIO:006007) | 3 |
|  | decision-making (BCIO:006116) | 11 |
|  | mental capability (MF:0000048) | 1 |
|  | planning (MF:0000027) | 2 |
| Motivation | appraisal of desirability of consequences (MFOEM:000085) | 1 |
|  | approval-based motivation (BCIO:006300) | 1 |
|  | craving (ADDICTO:0000511) | 1 |
|  | intrinsic motivation (BCIO:006136) | 1 |
|  | motivation (BCIO:006133) | 2 |
|  | pleasure associated with behaviour (BCIO:006159) | 1 |
|  | promotion focused motivational orientation (BCIO:006062) | 2 |
| Perceived Susceptibility /Vulnerability | appraisal (MFOEM:000005) | 1 |
|  | appraisal of dangerousness (MFOEM:000103) | 1 |
|  | belief about personal susceptibility (BCIO:006031) | 1 |
|  | belief about severity of an outcome (BCIO:006030) | 1 |
|  | belief about threat (BCIO:006306) | 6 |
| Reinforcement | appraisal of pleasantness (MFOEM:000061) | 1 |
|  | belief about anticipated emotion (BCIO:006140) | 3 |
|  | internal reward for a response (BCIO:006100) | 1 |
| Self-Image | evaluation of self (BCIO:006035) | 7 |
|  | self-identity (ADDICTO:0000399) | 15 |
|  | social identity (ADDICTO:0001087) | 3 |
| Skills | learning (BCIO:050239) | 1 |
|  | self-regulatory skill (BCIO:050222) | 1 |
| Social Influences | belief about social consequences of behaviour (BCIO:006020) | 1 |
|  | inter-personal behaviour (BCIO:036025) | 5 |
|  | linguistic communication behaviour (BCIO:050237) | 1 |
|  | non-linguistic communication behaviour (BCIO:050238) | 1 |
|  | social influence behaviour (BCIO:006099) | 2 |
| Values | personal value (BCIO:006063) | 1 |

Supplementary file 4

Entities from the MoA ontology co-occurring within a scale/subscale >5 times.

| **Combination** | **Mechanisms of action** | **n** |
| --- | --- | --- |
| 2 | self-regulation capability/self-regulation process | 36 |
| 2 | individual human behaviour/self-regulation process | 34 |
| 2 | behavioural self-regulation capability/self-regulation of behaviour | 28 |
| 2 | individual human behaviour/self-regulation of behaviour | 28 |
| 2 | behavioural self-regulation capability/individual human behaviour | 25 |
| 2 | individual human behaviour/self-regulation capability | 23 |
| 2 | self-regulation of behaviour/self-regulation process | 23 |
| 2 | emotional self-regulation capability/self-regulation process | 21 |
| 2 | behavioural self-regulation capability/self-regulation process | 19 |
| 2 | self-regulation capability/self-regulation of behaviour | 14 |
| 2 | behavioural self-regulation capability/self-regulation capability | 13 |
| 2 | decision-making/individual human behaviour | 12 |
| 2 | decision-making/self-regulation of behaviour | 12 |
| 2 | behavioural self-regulation capability/decision-making | 11 |
| 2 | decision-making/self-regulation process | 11 |
| 2 | emotion process/self-regulation process | 11 |
| 2 | emotional self-regulation capability/individual human behaviour | 11 |
| 2 | mental capability/self-regulation process | 11 |
| 2 | emotional self-regulation capability/self-regulation capability | 10 |
| 2 | individual human behaviour/mental capability | 10 |
| 2 | individual human behaviour/self-identity | 10 |
| 2 | linguistic communication behaviour/self-regulation process | 10 |
| 2 | self-identity/self-regulation process | 10 |
| 2 | action schema/self-regulation process | 9 |
| 2 | decision-making/self-regulation capability | 9 |
| 2 | goal pursuit process/individual human behaviour | 9 |
| 2 | mental capability/self-regulation of behaviour | 9 |
| 2 | self-identity/self-regulation capability | 9 |
| 2 | self-identity/self-regulation of behaviour | 9 |
| 2 | attentional self-regulation capability/self-regulation process | 8 |
| 2 | behavioural self-regulation capability/self-efficacy belief for a behaviour | 8 |
| 2 | behavioural self-regulation capability/self-identity | 8 |
| 2 | emotion process/individual human behaviour | 8 |
| 2 | emotion process/self-regulation capability | 8 |
| 2 | emotion process/self-regulation of behaviour | 8 |
| 2 | mental capability/self-regulation capability | 8 |
| 2 | self-efficacy belief for a behaviour/self-regulation of behaviour | 8 |
| 2 | action schema/self-regulation capability | 7 |
| 2 | appraisal of pleasantness/pleasure associated with behaviour | 7 |
| 2 | attentional self-regulation capability/individual human behaviour | 7 |
| 2 | behavioural self-regulation capability/emotional self-regulation capability | 7 |
| 2 | behavioural self-regulation capability/mental capability | 7 |
| 2 | emotional self-regulation capability/self-regulation of behaviour | 7 |
| 2 | goal pursuit process/self-regulation capability | 7 |
| 2 | goal pursuit process/self-regulation process | 7 |
| 2 | individual human behaviour/linguistic communication behaviour | 7 |
| 2 | individual human behaviour/self-efficacy belief for a behaviour | 7 |
| 2 | linguistic communication behaviour/self-regulation of behaviour | 7 |
| 2 | mental capability/self-efficacy belief for a behaviour | 7 |
| 2 | self-regulation process/thinking | 7 |
| 2 | action schema/individual human behaviour | 6 |
| 2 | attending/self-regulation process | 6 |
| 2 | behavioural self-regulation capability/emotion process | 6 |
| 2 | behavioural self-regulation capability/goal pursuit process | 6 |
| 2 | behavioural self-regulation capability/situational self-efficacy belief for a behaviour | 6 |
| 2 | belief/individual human behaviour | 6 |
| 2 | belief/self-regulation process | 6 |
| 2 | decision-making/goal pursuit process | 6 |
| 2 | decision-making/thinking | 6 |
| 2 | goal pursuit process/self-regulation of behaviour | 6 |
| 2 | individual human behaviour/planning | 6 |
| 2 | individual human behaviour/situational self-efficacy belief for a behaviour | 6 |
| 2 | individual human behaviour/thinking | 6 |
| 2 | mental capability/self-identity | 6 |
| 2 | self-efficacy belief for a behaviour/self-regulation capability | 6 |
| 2 | self-efficacy belief for a behaviour/self-regulation process | 6 |
| 2 | self-regulation capability/thinking | 6 |
| 2 | self-regulation of behaviour/situational self-efficacy belief for a behaviour | 6 |
| 3 | behavioural self-regulation capability/individual human behaviour/self-regulation of behaviour | 25 |
| 3 | individual human behaviour/self-regulation capability/self-regulation process | 23 |
| 3 | behavioural self-regulation capability/self-regulation of behaviour/self-regulation process | 19 |
| 3 | individual human behaviour/self-regulation of behaviour/self-regulation process | 19 |
| 3 | behavioural self-regulation capability/individual human behaviour/self-regulation process | 17 |
| 3 | self-regulation capability/self-regulation of behaviour/self-regulation process | 14 |
| 3 | behavioural self-regulation capability/self-regulation capability/self-regulation of behaviour | 13 |
| 3 | behavioural self-regulation capability/self-regulation capability/self-regulation process | 13 |
| 3 | individual human behaviour/self-regulation capability/self-regulation of behaviour | 13 |
| 3 | behavioural self-regulation capability/individual human behaviour/self-regulation capability | 12 |
| 3 | behavioural self-regulation capability/decision-making/self-regulation of behaviour | 11 |
| 3 | emotional self-regulation capability/individual human behaviour/self-regulation process | 11 |
| 3 | decision-making/individual human behaviour/self-regulation of behaviour | 10 |
| 3 | behavioural self-regulation capability/decision-making/individual human behaviour | 9 |
| 3 | decision-making/individual human behaviour/self-regulation process | 9 |
| 3 | decision-making/self-regulation capability/self-regulation process | 9 |
| 3 | decision-making/self-regulation of behaviour/self-regulation process | 9 |
| 3 | emotional self-regulation capability/self-regulation capability/self-regulation process | 9 |
| 3 | individual human behaviour/self-identity/self-regulation process | 9 |
| 3 | self-identity/self-regulation capability/self-regulation process | 9 |
| 3 | behavioural self-regulation capability/decision-making/self-regulation process | 8 |
| 3 | behavioural self-regulation capability/self-efficacy belief for a behaviour/self-regulation of behaviour | 8 |
| 3 | behavioural self-regulation capability/self-identity/self-regulation of behaviour | 8 |
| 3 | decision-making/individual human behaviour/self-regulation capability | 8 |
| 3 | individual human behaviour/mental capability/self-regulation of behaviour | 8 |
| 3 | individual human behaviour/mental capability/self-regulation process | 8 |
| 3 | individual human behaviour/self-identity/self-regulation capability | 8 |
| 3 | individual human behaviour/self-identity/self-regulation of behaviour | 8 |
| 3 | mental capability/self-regulation capability/self-regulation process | 8 |
| 3 | self-identity/self-regulation capability/self-regulation of behaviour | 8 |
| 3 | self-identity/self-regulation of behaviour/self-regulation process | 8 |
| 3 | action schema/self-regulation capability/self-regulation process | 7 |
| 3 | attentional self-regulation capability/individual human behaviour/self-regulation process | 7 |
| 3 | behavioural self-regulation capability/decision-making/self-regulation capability | 7 |
| 3 | behavioural self-regulation capability/emotional self-regulation capability/self-regulation of behaviour | 7 |
| 3 | behavioural self-regulation capability/emotional self-regulation capability/self-regulation process | 7 |
| 3 | behavioural self-regulation capability/individual human behaviour/self-efficacy belief for a behaviour | 7 |
| 3 | behavioural self-regulation capability/individual human behaviour/self-identity | 7 |
| 3 | behavioural self-regulation capability/mental capability/self-regulation of behaviour | 7 |
| 3 | behavioural self-regulation capability/self-identity/self-regulation capability | 7 |
| 3 | behavioural self-regulation capability/self-identity/self-regulation process | 7 |
| 3 | decision-making/self-regulation capability/self-regulation of behaviour | 7 |
| 3 | emotion process/self-regulation capability/self-regulation process | 7 |
| 3 | emotional self-regulation capability/self-regulation of behaviour/self-regulation process | 7 |
| 3 | goal pursuit process/individual human behaviour/self-regulation capability | 7 |
| 3 | goal pursuit process/individual human behaviour/self-regulation process | 7 |
| 3 | goal pursuit process/self-regulation capability/self-regulation process | 7 |
| 3 | individual human behaviour/self-efficacy belief for a behaviour/self-regulation of behaviour | 7 |
| 3 | linguistic communication behaviour/self-regulation of behaviour/self-regulation process | 7 |
| 3 | mental capability/self-regulation of behaviour/self-regulation process | 7 |
| 3 | action schema/individual human behaviour/self-regulation capability | 6 |
| 3 | action schema/individual human behaviour/self-regulation process | 6 |
| 3 | behavioural self-regulation capability/emotion process/individual human behaviour | 6 |
| 3 | behavioural self-regulation capability/emotion process/self-regulation of behaviour | 6 |
| 3 | behavioural self-regulation capability/emotional self-regulation capability/individual human behaviour | 6 |
| 3 | behavioural self-regulation capability/goal pursuit process/ individual human behaviour | 6 |
| 3 | behavioural self-regulation capability/goal pursuit process/self-regulation of behaviour | 6 |
| 3 | behavioural self-regulation capability/individual human behaviour/mental capability | 6 |
| 3 | behavioural self-regulation capability/individual human behaviour/situational self-efficacy belief for a behaviour | 6 |
| 3 | behavioural self-regulation capability/self-regulation of behaviour/situational self-efficacy belief for a behaviour | 6 |
| 3 | decision-making/goal pursuit process/individual human behaviour | 6 |
| 3 | emotion process/individual human behaviour/self-regulation capability | 6 |
| 3 | emotion process/individual human behaviour/self-regulation of behaviour | 6 |
| 3 | emotion process/individual human behaviour/self-regulation process | 6 |
| 3 | emotion process/self-regulation of behaviour/self-regulation process | 6 |
| 3 | emotional self-regulation capability/individual human behaviour/self-regulation of behaviour | 6 |
| 3 | goal pursuit process/individual human behaviour/self-regulation of behaviour | 6 |
| 3 | individual human behaviour/linguistic communication behaviour/self-regulation process | 6 |
| 3 | individual human behaviour/mental capability/self-identity | 6 |
| 3 | individual human behaviour/mental capability/self-regulation capability | 6 |
| 3 | individual human behaviour/self-regulation of behaviour/situational self-efficacy belief for a behaviour | 6 |
| 3 | individual human behaviour/self-regulation process/thinking | 6 |
| 3 | mental capability/self-identity/self-regulation process | 6 |
| 3 | mental capability/self-regulation capability/self-regulation of behaviour | 6 |
| 3 | self-efficacy belief for a behaviour/self-regulation capability/self-regulation process | 6 |
| 3 | self-regulation capability/self-regulation process/thinking | 6 |
| 4 | behavioural self-regulation capability/individual human behaviour/self-regulation of behaviour/self-regulation process | 17 |
| 4 | behavioural self-regulation capability/self-regulation capability/self-regulation of behaviour/self-regulation process | 13 |
| 4 | individual human behaviour/self-regulation capability/self-regulation of behaviour/self-regulation process | 13 |
| 4 | behavioural self-regulation capability/individual human behaviour/self-regulation capability/self-regulation of behaviour | 12 |
| 4 | behavioural self-regulation capability/individual human behaviour/self-regulation capability/self-regulation process | 12 |
| 4 | behavioural self-regulation capability/decision-making/individual human behaviour/self-regulation of behaviour | 9 |
| 4 | behavioural self-regulation capability/decision-making/self-regulation of behaviour/self-regulation process | 8 |
| 4 | decision-making/individual human behaviour/self-regulation capability/self-regulation process | 8 |
| 4 | individual human behaviour/self-identity/self-regulation capability/self-regulation process | 8 |
| 4 | self-identity/self-regulation capability/self-regulation of behaviour/self-regulation process | 8 |
| 4 | behavioural self-regulation capability/decision-making/self-regulation capability/self-regulation of behaviour | 7 |
| 4 | behavioural self-regulation capability/decision-making/self-regulation capability/self-regulation process | 7 |
| 4 | behavioural self-regulation capability/emotional self-regulation capability/self-regulation of behaviour/self-regulation process | 7 |
| 4 | behavioural self-regulation capability/individual human behaviour/self-efficacy belief for a behaviour/self-regulation of behaviour | 7 |
| 4 | behavioural self-regulation capability/individual human behaviour/self-identity/self-regulation of behaviour | 7 |
| 4 | behavioural self-regulation capability/self-identity/self-regulation capability/self-regulation of behaviour | 7 |
| 4 | behavioural self-regulation capability/self-identity/self-regulation capability/self-regulation process | 7 |
| 4 | behavioural self-regulation capability/self-identity/self-regulation of behaviour/self-regulation process | 7 |
| 4 | decision-making/individual human behaviour/self-regulation of behaviour/self-regulation process | 7 |
| 4 | decision-making/self-regulation capability/self-regulation of behaviour/self-regulation process | 7 |
| 4 | goal pursuit process/individual human behaviour/self-regulation capability/self-regulation process | 7 |
| 4 | individual human behaviour/mental capability/self-regulation of behaviour/self-regulation process | 7 |
| 4 | individual human behaviour/self-identity/self-regulation capability/self-regulation of behaviour | 7 |
| 4 | individual human behaviour/self-identity/self-regulation of behaviour/self-regulation process | 7 |
| 4 | action schema/individual human behaviour/self-regulation capability/self-regulation process | 6 |
| 4 | behavioural self-regulation capability/decision-making/individual human behaviour/self-regulation capability | 6 |
| 4 | behavioural self-regulation capability/decision-making/individual human behaviour/self-regulation process | 6 |
| 4 | behavioural self-regulation capability/emotion process/individual human behaviour/self-regulation of behaviour | 6 |
| 4 | behavioural self-regulation capability/emotional self-regulation capability/individual human behaviour/self-regulation of behaviour | 6 |
| 4 | behavioural self-regulation capability/emotional self-regulation capability/individual human behaviour/self-regulation process | 6 |
| 4 | behavioural self-regulation capability/goal pursuit process/individual human behaviour/self-regulation of behaviour | 6 |
| 4 | behavioural self-regulation capability/individual human behaviour/mental capability/self-regulation of behaviour | 6 |
| 4 | behavioural self-regulation capability/individual human behaviour/self-identity/self-regulation capability | 6 |
| 4 | behavioural self-regulation capability/individual human behaviour/self-identity/self-regulation process | 6 |
| 4 | behavioural self-regulation capability/individual human behaviour/self-regulation of behaviour/situational self-efficacy belief for a behaviour | 6 |
| 4 | decision-making/individual human behaviour/self-regulation capability/self-regulation of behaviour | 6 |
| 4 | emotion process/individual human behaviour/self-regulation capability/self-regulation process | 6 |
| 4 | emotional self-regulation capability/individual human behaviour/self-regulation of behaviour/self-regulation process | 6 |
| 4 | individual human behaviour/mental capability/self-identity/self-regulation process | 6 |
| 4 | individual human behaviour/mental capability/self-regulation capability/self-regulation of behaviour | 6 |
| 4 | individual human behaviour/mental capability/self-regulation capability/self-regulation process | 6 |
| 4 | mental capability/self-regulation capability/self-regulation of behaviour/self-regulation process | 6 |
| 5 | behavioural self-regulation capability/individual human behaviour/self-regulation capability/self-regulation of behaviour/self-regulation process | 12 |
| 5 | behavioural self-regulation capability/decision-making/self-regulation capability/self-regulation of behaviour/self-regulation process | 7 |
| 5 | behavioural self-regulation capability/self-identity/self-regulation capability/self-regulation of behaviour/self-regulation process | 7 |
| 5 | individual human behaviour/self-identity/self-regulation capability/self-regulation of behaviour/self-regulation process | 7 |
| 5 | behavioural self-regulation capability/decision-making/individual human behaviour/self-regulation capability/self-regulation of behaviour | 6 |
| 5 | behavioural self-regulation capability/decision-making/individual human behaviour/self-regulation capability/self-regulation process | 6 |
| 5 | behavioural self-regulation capability/decision-making/individual human behaviour/self-regulation of behaviour/self-regulation process | 6 |
| 5 | behavioural self-regulation capability/emotional self-regulation capability/individual human behaviour/self-regulation of behaviour/self-regulation process | 6 |
| 5 | behavioural self-regulation capability/individual human behaviour/self-identity/self-regulation capability/self-regulation of behaviour | 6 |
| 5 | behavioural self-regulation capability/individual human behaviour/self-identity/self-regulation capability/self-regulation process | 6 |
| 5 | behavioural self-regulation capability/individual human behaviour/self-identity/self-regulation of behaviour/self-regulation process | 6 |
| 5 | decision-making/individual human behaviour/self-regulation capability/self-regulation of behaviour/self-regulation process | 6 |
| 5 | individual human behaviour/mental capability/self-regulation capability/self-regulation of behaviour/self-regulation process | 6 |
| 6 | behavioural self-regulation capability/decision-making/individual human behaviour/self-regulation capability/self-regulation of behaviour/self-regulation process | 6 |
| 6 | behavioural self-regulation capability/individual human behaviour/self-identity/self-regulation capability/self-regulation of behaviour/self-regulation process | 6 |
